# Effects of positive end-expiratory pressure on regional cerebral oxygen saturation in elderly patients undergoing thoracic surgery during one-lung ventilation: a randomized crossover-controlled trial

**DOI:** 10.1186/s12890-024-02931-z

**Published:** 2024-03-06

**Authors:** Liying Zhao, Shuang Lv, Qian Xiao, Yuan Zhang, Wenbo Yi, Yu Bai, Kangping Lu, Kevin C. Bermea, Jessica Semel, Xiaomei Yang, Jianbo Wu

**Affiliations:** 1 Department of Anesthesiology, Qilu Hospital of Shandong University, 107 #, Wenhua Xi Road, 250012 Jinan, Shandong China; 2 School of Medicine, Cheeloo College of Medicine, Shandong University, 250012 Jinan, China; 3 Central Hospital of Enshi Tujia and Miao Autonomous Prefecture, 445000 Enshi City, Hubei Province China; 4 Department of Anesthesiology and Perioperative Medicine, Shandong Institute of Anesthesia and Respiratory Critical Medicine, The First Affiliated Hospital of Shandong First Medical University & Shandong Provincial Qianfoshan Hospital, 16766 #, Jingshi Road, 250012 Jinan, Shandong China; 5 Clinical Epidemiology Unit, Qilu Hospital of Shandong University, 250012 Jinan, Shandong China; 6 Department of Medicine, Division of Cardiology, Johns Hopkins University School of Medicine, 21205 Baltimore, MD USA; 7 Department of Biochemistry and Molecular Biology, Center for Research on Cardiac Intermediate Filaments, Johns Hopkins University School of Medicine, 21205 Baltimore, MD USA

**Keywords:** Hemodynamics, One-lung ventilation, Positive end-expiratory pressure, Pulmonary oxygenation, Regional cerebral oxygen saturation

## Abstract

**Background:**

A significant reduction in regional cerebral oxygen saturation (rSO_2_) is commonly observed during one-lung ventilation (OLV), while positive end-expiratory pressure (PEEP) can improve oxygenation. We compared the effects of three different PEEP levels on rSO_2_, pulmonary oxygenation, and hemodynamics during OLV.

**Methods:**

Forty-three elderly patients who underwent thoracoscopic lobectomy were randomly assigned to one of six PEEP combinations which used a crossover design of 3 levels of PEEP—0 cmH_2_O, 5 cmH_2_O, and 10 cmH_2_O. The primary endpoint was rSO_2_ in patients receiving OLV 20 min after adjusting the PEEP. The secondary outcomes included hemodynamic and respiratory variables.

**Results:**

After exclusion, thirty-six patients (36.11% female; age range: 60–76 year) were assigned to six groups (*n* = 6 in each group). The rSO_2_ was highest at OLV(0) than at OLV(10) (difference, 2.889%; [95% CI, 0.573 to 5.204%]; *p* = 0.008). Arterial oxygen partial pressure (PaO_2_) was lowest at OLV(0) compared with OLV(5) (difference, -62.639 mmHg; [95% CI, -106.170 to -19.108 mmHg]; *p* = 0.005) or OLV(10) (difference, -73.389 mmHg; [95% CI, -117.852 to -28.925 mmHg]; *p* = 0.001), while peak airway pressure (Ppeak) was lower at OLV(0) (difference, -4.222 mmHg; [95% CI, -5.140 to -3.304 mmHg]; *p* < 0.001) and OLV(5) (difference, -3.139 mmHg; [95% CI, -4.110 to -2.167 mmHg]; *p* < 0.001) than at OLV(10).

**Conclusions:**

PEEP with 10 cmH_2_O makes rSO_2_ decrease compared with 0 cmH_2_O. Applying PEEP with 5 cmH_2_O during OLV in elderly patients can improve oxygenation and maintain high rSO_2_ levels, without significantly increasing peak airway pressure compared to not using PEEP.

**Trial registration:**

Chinese Clinical Trial Registry ChiCTR2200060112 on 19 May 2022.

**Supplementary Information:**

The online version contains supplementary material available at 10.1186/s12890-024-02931-z.

## Introduction

An estimated account for 2 million new cases and 1.76 million deaths due to lung cancer each year [1]. Video-assisted thoracoscopic lobectomy has obvious advantages with smaller invasion, faster postoperative recovery, and fewer postoperative complications, especially in the elderly or those with poor lung function or comorbidities [2]. One-lung ventilation (OLV) can improve the quality of the operation field and accelerate the process of operation, so OLV is used in almost all surgeries now performed on the lung or in thoracic surgeries that rely on lung collapse to provide optimal surgical exposure [3].

A collapsed, unventilated lung causes an increscent shunt fraction and ventilation-perfusion (V/Q) mismatch during OLV, so hypoxemia may occur [3–5]. Arterial hypoxemia occurs in 9–27% of patients receiving OLV [6]. OLV may also cause postoperative pulmonary complications (PPCs), especially in elderly individuals, and complications are reported to occur in 7.3–13.3% of patients [7]. Positive end-expiratory pressure (PEEP) as part of the perioperative lung protection ventilation strategy (LPVS) has been gaining popularity because it can improve oxygenation by optimizing respiratory system mechanics, maintaining functional residual capacity, and aiding in alveolar recruitment [8]. However, higher PEEP has been affiliated with exceeding intrathoracic pressure, reducing venous return, and eventually lessening cardiac output (CO).

A significant reduction in regional cerebral oxygen saturation (rSO_2_) is commonly observed following thoracic surgery during OLV [9–11]. The measurement of arterial oxygen partial pressure (PaO_2_) or peripheral oxygen saturation does not provide enough information to detect significant cerebral oxygen desaturation [10]. Older patients are more prone to developing cerebral desaturation than younger patients because of reduced respiratory physiological reserve and the presence of coexisting diseases. Findings by Casati suggested that older patients may suffer less postoperative cognitive decline as well as shorter hospital stays by monitoring rSO_2_ to detect brain desaturation and subsequently improve arterial oxygen saturation [12].

Although it is generally believed that proper PEEP is beneficial for oxygenation during OLV, whether PEEP could enhance rSO_2_ is uncertain. There have been studies showing the change in rSO_2_ during OLV, but the relationship between PEEP and rSO_2_ in the elderly population during OLV has not been studied. Based on the above studies, the purpose of the study was to observe the effects of different PEEP levels on rSO_2_, pulmonary oxygenation, and hemodynamics in elderly patients who underwent thoracoscopic lobectomy during OLV. In addition, a suitable PEEP value is needed to enhance oxygenation at the utmost and minimize damage to peak airway pressure (Ppeak) and hemodynamics in elderly patients undergoing thoracic surgery.

## Materials and methods

### Ethics

Institutional Review Board approval was obtained before the study. The study protocol was approved by the Ethics Committee of Scientific Research of Qilu Hospital of Shandong University. It was also registered with the Chinese Clinical Trial Registry (ChiCTR2200060112) on 19 May 2022.

### Patients

This study was conducted from June 2022 to October 2022 at a tertiary A medical center. Forty-three elderly patients who underwent elective thoracoscopic lobectomy were selected. Subjects were visited one day before the operation to learn about their general conditions. Before randomization and study procedures, signed informed consent was obtained. Patients who met the following inclusion criteria were included: (1) aged between 60 and 80 years; (2) American Society of Anesthesiologists (ASA) physical status I-III; and (3) underwent elective thoracoscopic lobectomy and required OLV. The exclusion criteria included the following: (1) had severe cardiovascular and cerebrovascular diseases; (2) had severe liver and kidney dysfunction or coagulation disorders; (3) had diseases for which cerebral oxygen saturation monitoring could not be performed, such as head deformity and forehead skin infection; and (4) had contraindications for radial artery puncture.

### Anesthesia and procedural protocols

After the patient entered the operating room, non-invasive arterial pressure, electrocardiogram, and saturation of peripheral oxygen (SpO_2_) were routinely monitored. After local anesthesia, radial artery puncture and catheterization were performed to measure arterial blood pressure. The electrodes of the adult brain oxygen saturation monitor were placed 1 cm from the middle of the forehead and 1–2 cm above the eyebrow arch. The rSO_2_ was then monitored using the INVOS 5100 C cerebral oxygen saturation monitor produced by Covidien LLC. Use the FloTrac/Vigileo apparatus to monitor cardiac index (CI) and stroke volume variability (SVV). Anesthesia was induced using midazolam (0.02–0.03 mg/kg), sufentanil (0.4–0.5 µg/kg), etomidate (0.2–0.3 mg/kg), and rocuronium bromide (0.6 mg/kg). After 3–5 min, an experienced anesthesiologist used a video laryngoscope for tracheal intubation, and then a bronchial occluder was placed on the affected side.

Anesthesia was subsequently maintained with 1.0–2.0 vol % of sevoflurane, 1–2 mg/(kg·h) of propofol, and 0.02–0.05 µg/(kg·min) of remifentanil aimed at obtaining a Bispectral index (BIS) between 40 and 60. Add rocuronium when needed. All subjects received volume-controlled ventilation (VCV) via the following parameters: tidal volume (Vt) 6–8 ml/kg predicted body weight (PBW); respiratory rate (RR) 12–14/min; 1:2 inspiratory-to-expiratory ratio (I: E); and 1.5 L/min oxygen flow. A fraction of inspiration oxygen (FiO_2_) of 1 was used to avoid hypoxemia and the influence of oxygen concentration on arterial blood gas results during OLV. Adjust ventilation parameters to maintain an end-tidal carbon dioxide (P_ET_CO_2_) pressure of 35-45mmHg.

### Study design

A prospective randomized crossover-controlled method was used in this study. For each subject, three different levels were applied successively— 0 cmH_2_O, 5 cmH_2_O, and 10 cmH_2_O—and the measurements of each PEEP application were designated OLV (0), OLV (5), and OLV(10), respectively, for statistical analysis. Patients were randomly assigned to a PEEP sequence combination of six (Supplemental Methods and eFigure 1 in the Supplement).

### Randomization and blinding

An independent investigator wrote the computer-generated randomization list. Randomization was conducted by hermetic, sequentially numbered, and non-transparent envelopes placed in the operating room.

Patients and investigators overseeing the study outcomes were blinded to group assignment. It was, however, not blinded to the attending anesthesiologists and intraoperative assessors.

### Data collection

The data was collected using a standardized case report form. Note subject characteristics and other information. During the intraoperative period, all vital signs and ventilatory data were recorded. The bilateral rSO_2_, heart rate (HR), SpO_2_, Ppeak, mean arterial pressure (MAP), CI, SVV, and arterial blood gas values were measured. Because vascular clamping can change the perfusion of the non-dependent lung, subjects were excluded from the experiment if the pulmonary vessel was clamped for lobectomy during the experiment. (Supplemental Methods in the Supplement)

### Study outcomes

The primary outcome was defined as the average of bilateral rSO_2_ among three different PEEPs at 20 min after adjusting every PEEP during OLV. And the secondary outcomes were rSO_2_ between OLV and two-lung ventilation (TLV), and respiratory variables such as PaO_2_, hemodynamics, and other parameters (Supplemental Methods in the Supplement). The rSO_2_ <65% was seen as a threshold that showed the increased risk of postoperative complications [13].

### Sample size estimation

The estimation of sample size was performed with PASS 15. Based on previous findings [14, 15] and taking the tripartite crossover clinical trial into account, the sample size was derived from the mean average rSO_2_ in the previous pilot trial (70.5, 67.0, and 65.6, respectively, with a standard deviation of 7.5) among the three PEEP groups. We used the Geisser-Greenhouse F Test with K = 1 and $\rho =$0.2. At the 5% significance level, 36 patients were required to provide a power value of 80% at a two-sided significance level of *p* = 0.05; assuming a dropout rate of 10%, 40 patients were needed at least.

### Statistical analysis

SPSS 25.0 statistical software was used for analysis. Categorical data are expressed as counts (proportion). Continuous variables are expressed as mean$\pm$standard deviation (SD) or median (interquartile range), depending on the normality distribution of the data assessed by the Shapiro-Wilk test and a histogram. The homogeneity of variances was verified with the Levene test. Data conforming to the normal distribution and homogeneity of variances were compared using repeated-measures ANOVA. The Greenhouse-Geisser correction was used if the data did not conform to spherical symmetry. If the results were statistically significant, Bonferroni analysis was conducted and used for pairwise comparisons. Data which not conform to the normal distribution or homogeneity of variances were analyzed using a generalized linear mixed model. We conducted a chi-square test to analyze the difference in rSO_2_ levels under 65% among the three PEEP groups. A *p-value* < 0.05 was considered statistically significant.

Before the formal analysis, we calculated the residuals of different PEEPs to verify the rationality of the statistics. Inference of the presence of PEEP residuals in different orders was performed by one-way ANOVA or Mann-Whitney rank-sum analysis with the group effect sum. If residuals were present, differences were assessed based on the data of PEEP after the first adjustment only. Otherwise, we used the statistical methods described above.

## Results

**Fig. 1:**
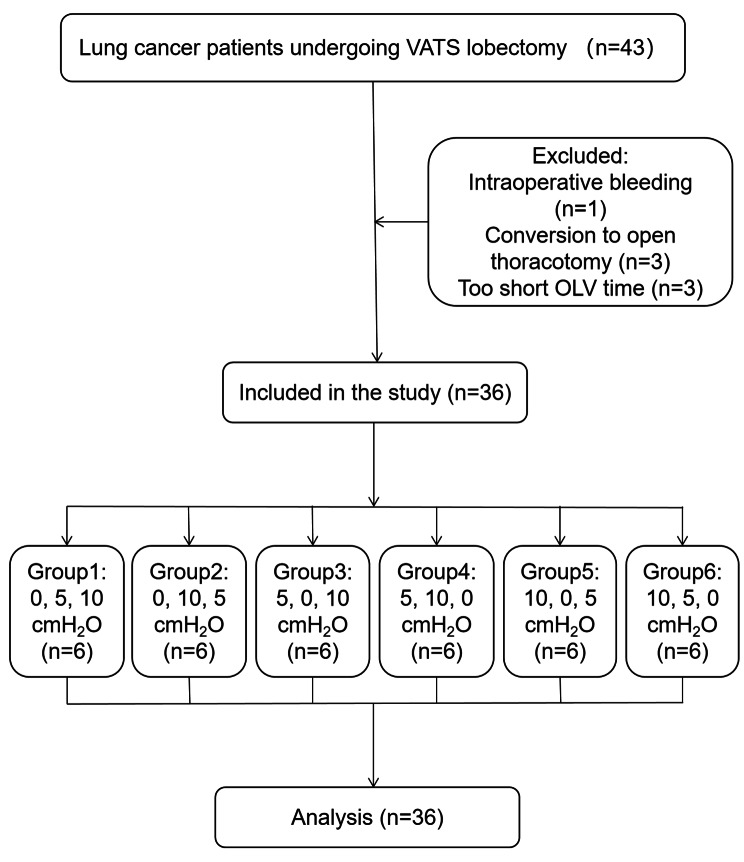
Flow Diagram of Patients Through Trial. VATS, video-assisted thoracoscopic surgery

Forty-three subjects were enrolled and randomized. Seven patients were excluded. One was due to intraoperative bleeding, three were due to conversion to open thoracotomy, and three were owing to too short OLV time (< 60 min), respectively. Thus, 36 subjects were included in the final analysis (Fig. 1). The baseline characteristics of the subjects are presented in Table 1. The overall mean age was 65.86 years, and the majority of the participants (*n* = 23 [63.89%]) were male. A total of 63.89% of participants underwent surgery on the right lung.

**Table 1:** Demographic characteristics and intraoperative data

	Patients (*n* = 36)
Age, mean ± SD, %,years	65.86 ± 4.33
Sex (male/female), [n (%)]	23(63.89)/13(36.11)
Height, mean ± SD, %, cm	162.03 ± 7.11
Weight, mean ± SD, %, Kg	65.14 ± 7.98
Lesion of the lung (left/right), [n (%)]	13(36.11)/23(63.89)
ASA (II/III), [n (%)]	29(80.56)/7(19.44)
FEV1, mean ± SD, %	109.94 ± 20.36
FEV1/FVC, mean ± SD, %	99.79 ± 8.26
rSO2, mean ± SD, %	69.46 ± 5.78
Duration of surgery, mean ± SD, %, min	131.17 ± 39.85
Duration of TLV, mean ± SD, %, min	50.89 ± 14.80
Duration of OLV, mean ± SD, %, min	117.89 ± 37.02
Preoperative Hb, mean ± SD, g/L	120.89 ± 13.06
Postoperative Hb, mean ± SD, g/L	117.25 ± 12.98
Intraoperative infusion volume (mL),median(IQR)	2000.0(1500.0,2000.0)
Urine output, mean ± SD, %, mL	340.3 ± 118.8
Estimated blood loss, mean ± SD, %, mL	26.8 ± 8.0

**Notes**: Data are presented as mean$\pm$standard deviation (SD), counts (proportion), or median(IQR). **Abbreviations**: ASA, American Society of Anesthesiologists; FEV1, forced expiratory volume in one second; FVC, forced vital capacity; TLV, two-lung ventilation; OLV, one-lung ventilation; Hb hemoglobin

After analysis, there was no significant difference in the PEEP order among the groups except for arterial carbon dioxide partial pressure (PaCO_2_) and HR (eTable 1 in the Supplement). Therefore, PaCO_2_ and HR were compared by one-way ANOVA based on the first-stage data of PEEP. Others used repeated measures ANOVA or a generalized linear mixed model with all six groups.

### Effect of PEEP on rSO_2_ in elderly patients during thoracoscopic lobectomy

**Fig. 2:**
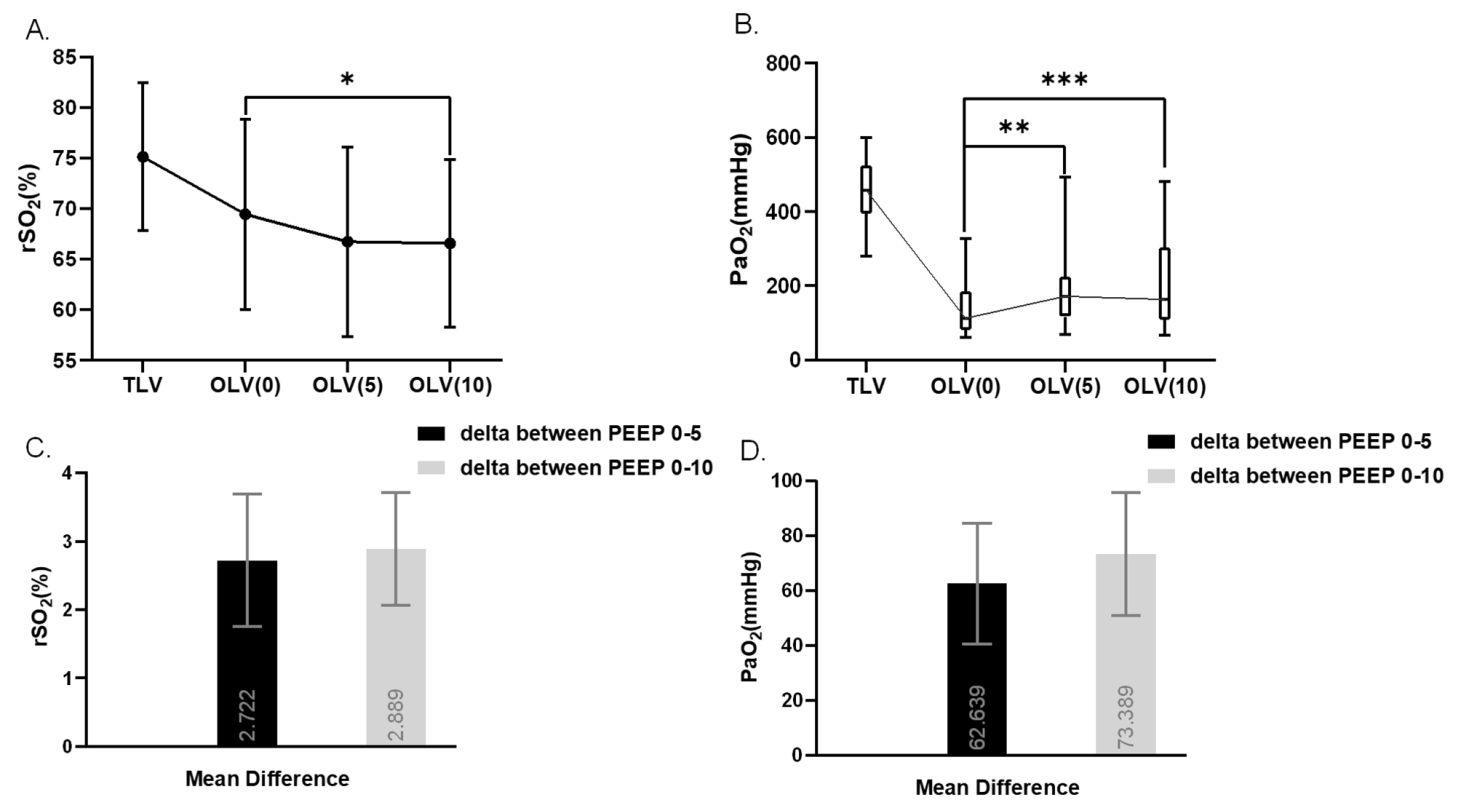
Effect of PEEP on rSO_2_ and PaO_2_ in elderly patients during thoracoscopic lobectomy. **(A.) (B.)**There were significant differences among TLV and three PEEP levels of OLV both in rSO_2_ and PaO_2_. * OLV(0) vs. OLV(10): *p* = 0.008. ** OLV(0) vs. OLV(5): *p* = 0.005;*** OLV(0) vs. OLV(10): *p* = 0.001; **(C.) (D.)** In the column chart, rectangles depict the mean differences, and the error bars signify the standard error. TLV, two-lung ventilation; OLV, one-lung ventilation; rSO_2_, regional cerebral oxygen saturation; PaO_2_, arterial oxygen partial pressure; OLV(0), OLV ventilation with 0 cmH_2_O PEEP; OLV(5), OLV ventilation with 5 cmH_2_O PEEP; OLV(10), OLV ventilation with 10 cmH_2_O PEEP

**Fig. 3:**
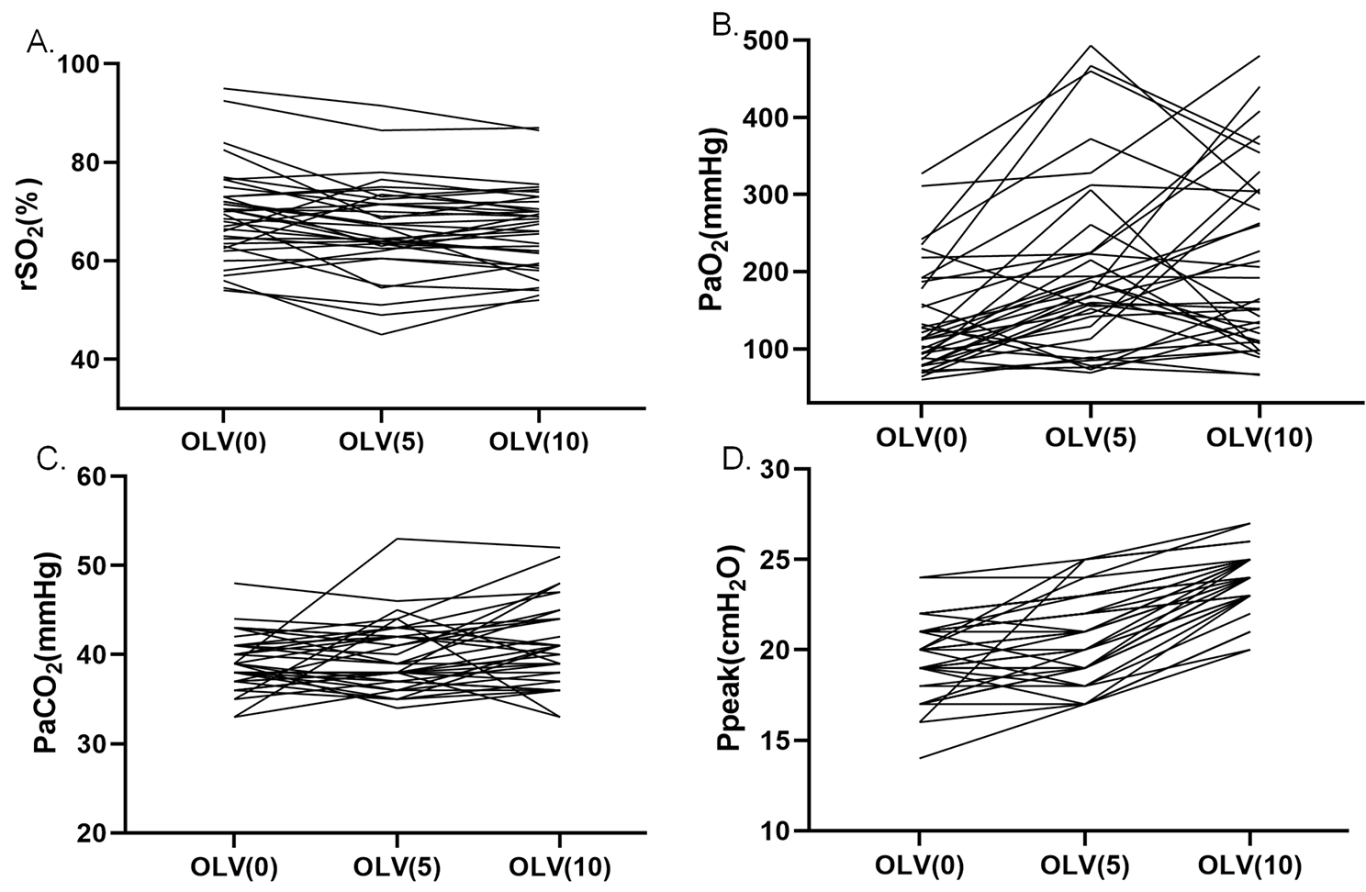
Effects of PEEP on rSO_2_, blood gas analysis, and respiratory variables during thoracoscopic lobectomy. **(A)** rSO_2_, regional cerebral oxygen saturation; **(B)** PaO_2_, arterial oxygen partial pressure; **(C)** PaCO_2_, arterial carbon dioxide partial pressure; **(D)** Ppeak, peak airway pressure. OLV, one-lung ventilation; OLV(0), OLV ventilation with 0 cmH_2_O PEEP; OLV(5), OLV ventilation with 5 cmH_2_O PEEP; OLV(10), OLV ventilation with 10 cmH_2_O PEEP

The rSO_2_ was significantly different among three PEEP levels during OLV (*p* = 0.002) (Table 2; Figs. 2 and 3, and eTable 2 in the Supplement). According to the pairwise comparisons, the rSO_2_ was greater at OLV(0) than OLV(10) (difference, 2.889%; [95% CI, 0.573 to 5.204%]; *p* = 0.008), but there was no statistical difference between OLV(0) and OLV(5) (*p* = 0.053), as well as between OLV(5) and OLV(10) (*p* > 0.999) (eTable 3 in the Supplement, Fig. 2). When switched from TLV to OLV, rSO_2_ was significantly decreased (*p* < 0.001) (Table 2). The results of pairwise comparisons are shown in eTable 3 in the Supplement. We compared the number of patients whose rSO_2_ was lower than 65% during OLV and found that there was no statistical difference among the three PEEP groups (*p* = 0.349) (eTable 4 in the Supplement).

**Table 2:** Effects of PEEP on regional cerebral oxygen saturation, ventilatory and hemodynamic data in elderly patients

Outcome	TLV	OLV			*P* value
		OLV (0)	OLV (5)	OLV (10)	
Primary outcome					
rSO_2_, mean ± SD, %	—	69 ± 9	67 ± 9	67 ± 8	0.002^b^
Secondary outcomes					
rSO_2_, mean ± SD, %	75 ± 7	69 ± 9	67 ± 9	667 ± 8	<0.001^b^
PaO_2_^a^,median(IQR),(mmHg)	—	112.0(80.6,184.6)	171.5(117.0,224.8)	163.0(109.3,303.3)	0.001
	457.5(394.0,523.5)				<0.001
PaCO_2_, mean ± SD, (mmHg)	—	39 ± 3	40 ± 4	41 ± 5	<0.001^c^
	39 ± 6				0.003^c^
Ppeak, mean ± SD,(cmH_2_O)	—	20 ± 2	21 ± 2	24 ± 2	<0.001
	16 ± 2				<0.001
HR, mean ± SD, (beats/min)	—	68 ± 13	62 ± 10	62 ± 11	0.140^c^
	67 ± 11				0.156^c^
MAP,median(IQR),(mmHg)	—	79.0(73.3,90.8)	82.67(76.5,91.5)	83.33(77.1,89.3)	0.442
	84.5(76.4,95.0)				0.452
CI,median(IQR),(L/min∙m^2^)	—	2.15(1.83,2.40)	2.15(1.83,2.30)	2.10(1.93,2.28)	0.894
	2.10(1.90,2.30)				0.961
SVV,median(IQR),%	—	9.00(5.00,10.00)	6.00(5.25,9.00)	7.00(5.25,9.00)	0.492
	9.00(8.00,11.00)				0.004

**Notes**: Data are presented as mean ± standard deviation (SD) or median (IQR). “-” means not participating in the statistical analysis of this row. ^a^. PaO_2_ values are equal to PaO_2_ / FiO_2_ values because of FiO_2_ = 100% in this study. ^b^. Use repeated measures ANOVA to analyze the data. Others used the generalized linear mixed model. ^c^. Use one-way ANOVA based on the first stage data of PEEP. **Abbreviations**: rSO_2_, regional cerebral oxygen saturation. TLV, two-lung ventilation; OLV, one-lung ventilation; PaO_2_, arterial oxygen partial pressure; PaCO_2_, arterial carbon dioxide partial pressure; Ppeak, peak airway pressure; HR, heart rate; MAP, mean arterial pressure; CI, cardiac index; SVV, stroke volume variability

### Influence of PEEP on arterial blood gas analysis and respiratory variables in elderly patients during OLV

PaO_2_ and PaO_2_ / FiO_2_ values were significantly different among the three PEEP levels in OLV (*p* = 0.001) (Table 2; Fig. 2, and Fig. 3). PaO_2_ and PaO_2_ / FiO_2_ values were lowest at OLV(0) compared with OLV(5) (difference, -62.639 mmHg; [95% CI, -106.170 to -19.108 mmHg]; *p* = 0.005) or OLV(10) (difference, -73.389 mmHg; [95% CI, -117.852 to -28.925 mmHg]; *p* = 0.001) according to pairwise comparisons, and there was no significant difference between OLV(5) and OLV(10) (difference, -10.750; [95% CI, -63.957 to 42.457]; *p* = 0.690) (eTable 3 in the Supplement).

No statistical differences in PaCO_2_ between OLV(0) and OLV(5) (difference, -0.583; [95% CI, -5.913 to 4.746]; *p* > 0.999) (eTable 3, Fig. 3 and eFigure 2 in the Supplement). But PaCO_2_ at OLV(0) was statistically lower than OLV(10) (difference, -6.333; [95% CI, -11.663 to -1.004]; *p* = 0.011), as well as at OLV(5) compared with OLV(10) (difference, -5.750; [95% CI, -11.079 to -0.420]; *p* = 0.027) (eTable 3 in the Supplement).

The data of Ppeak were significantly different among the three levels of PEEP during OLV (*p* < 0.001) (Table 2; Fig. 3, and eFigure 2 in the Supplement). Ppeak at OLV(0) (difference, -4.222 mmHg; [95% CI, -5.140 to -3.304 mmHg]; *p* < 0.001) and OLV(5) (difference, -3.139 mmHg; [95% CI, -4.110 to -2.167 mmHg]; *p* < 0.001) were statistically lower than OLV(10), while no significant difference was found between OLV(0) and OLV (5) (difference, -1.083; [95% CI, -2.170 to 0.003]; *p* = 0.051) (eTable 3 in the Supplement).

### Effect of PEEP on hemodynamics in elderly patients during OLV

**Fig. 4:**
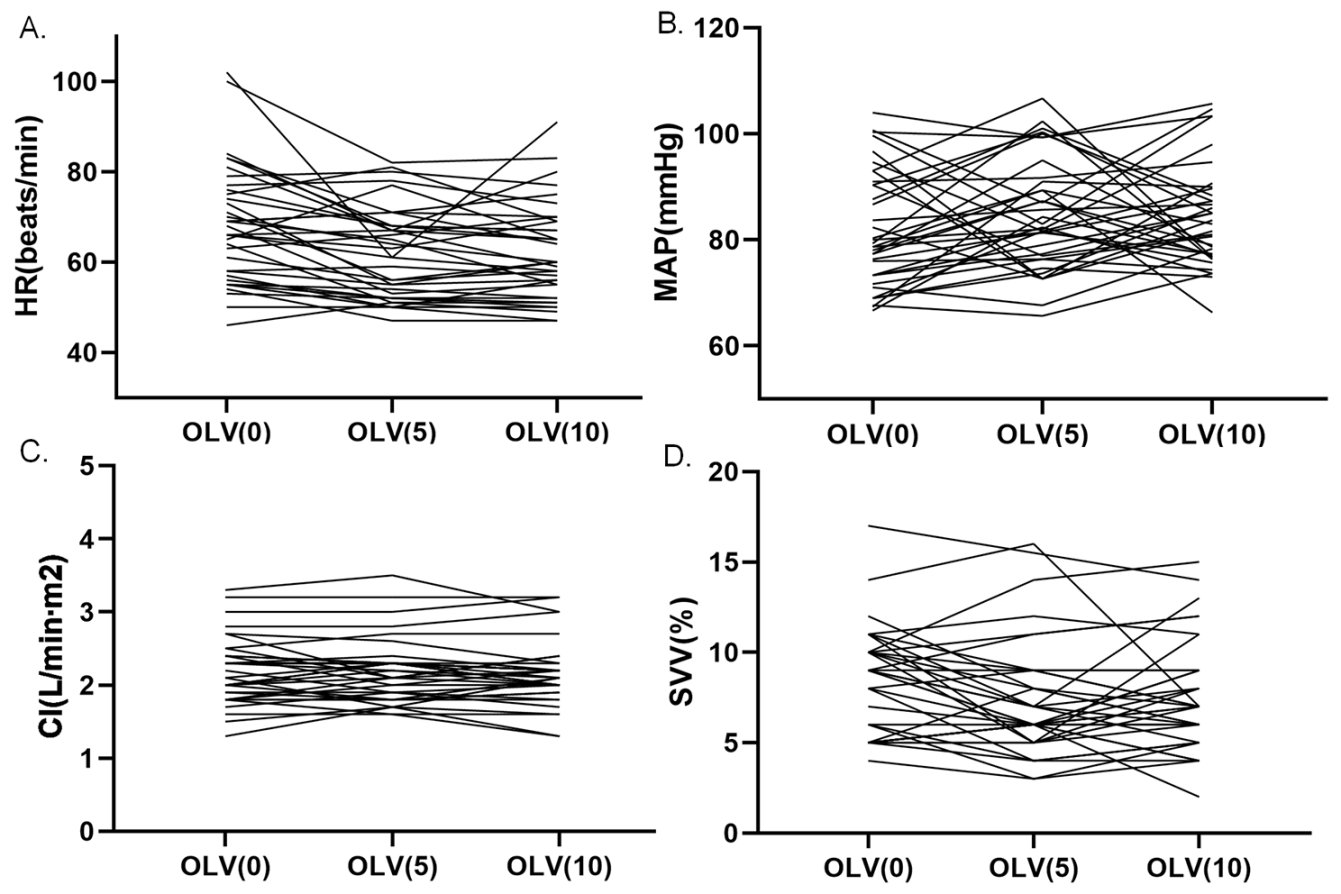
Effects of PEEP on hemodynamics during thoracoscopic lobectomy. **(A)** HR, heart rate; **(B)** MAP, mean arterial pressure; **(C)** CI, cardiac index; **(D)** SVV, stroke volume variability. OLV, one-lung ventilation; OLV(0), OLV ventilation with 0 cmH_2_O PEEP; OLV(5), OLV ventilation with 5 cmH_2_O PEEP; OLV(10), OLV ventilation with 10 cmH_2_O PEEP

No significant difference was observed in stroke volume variability (SVV) during OLV under any of the PEEP conditions (*p* = 0.492), while it was lower during OLV than during TLV (*p* = 0.004) (Table 2, eFigure 2 in the Supplement). There were no statistically significant differences in HR, MAP, or CI between the TLV and OLV, or among different PEEP groups (Table 2; Fig. 4, and eFigure 2 in the Supplement).

## Discussion

In this study, the rSO_2_ data were significantly decreased during OLV in 10 cmH_2_O PEEP compared with 0 cmH_2_O, suggesting that the rSO_2_ could be affected as the PEEP increased. Fewer studies have demonstrated the relationship between rSO_2_ and PEEP in elderly patients during OLV. The rSO_2_ reflects changes in brain tissue oxygenation and blood flow [16, 17]. In theory, a high PEEP may increase intracranial pressure (ICP) by increasing central venous pressure (CVP) [18, 19], resulting in a reduced cerebral perfusion pressure (CPP), which may be one of the reasons why the rSO_2_ decreased. However, a study showed that in neurological and neurosurgical patients, the application of a high PEEP (at 10–15 cmH_2_0) increased ICP significantly, with no significant change in CPP [20]. A study by FROST et al. [21] found that in patients with normal or lower intracranial compliance, 40 cmH_2_O of PEEP did not increase the ICP. Therefore, the decrease in rSO_2_ in this study may be related to the changed ICP, but the reason is still controversial because rSO_2_ could be affected by some other factors like CO and Hb. CI was chosen over CO for this experiment, as it ruled out the effect of the patient’s body size. There was no significant difference in the patient’s hemodynamic parameters among the groups. We also analyzed the patients’ preoperative and postoperative hemoglobin data, and there was no statistical difference (120.89 ± 13.06 g/L vs. 117.25 ± 12.98 g/L, *p* = 0.240).

In addition, Kazan [11] thought when the absolute value of the rSO_2_ decreased to less than 65%, postoperative morbidity would rise. In this study, the mean values of rSO_2_ at 0, 5, and 10 cmH_2_O were all greater than 65% (69.46%, 66.74%, and 66.57%). So, the PEEP within 10 cmH_2_O used in this study during OLV is acceptable. Further prospective research is needed to evaluate the safety and clinical effects of applying PEEP to a different range of patients.

The incidence of a decrease with rSO_2_ was significantly higher after thoracic surgery. Kazan et al. [11]proved that 82% of patients had at least a 15% decrease in rSO_2_ from the baseline value among the 50 patients who underwent thoracic surgery. This study was consistent with the above conclusion, showing that the rSO_2_ decreased markedly during OLV compared with TLV, indicating that OLV may be associated with decreased cerebral oxygen delivery and utilization in thoracic surgery. The rSO_2_ reflects the relationship between oxygen supply and oxygen consumption, which are impacted by lots of factors like age, temperature, arterial oxygen saturation (SaO_2_), CO, cerebral blood flow (CBF), Hb, and so on [19, 20]. OLV can cause intrapulmonary shunting and V/Q mismatch, meanwhile, it can cause hemodynamic changes by ventilatory pressure and increasing pulmonary vascular resistance caused by hypoxic pulmonary vasoconstriction [22, 23]. Meng et al. [24] showed that changes in cerebral oxygenation may be related to changes in CO. Thus, the decrease in rSO_2_ in this study may be caused by the reduction in oxygen supply, which is not only related to SaO_2_ but also related to Hb and CO, especially in the elderly population.

This trial found that PaO_2_ was reduced by over 60% when switched from TLV to OLV at 0 cmH_2_0 PEEP, which is similar to the findings of Ferrando et al. [25]. However, the specific impacts of PEEP on arterial oxygenation and ventilatory mechanics during OLV in the elderly remain uncertain. It is currently recognized that zero PEEP has a negative impact [26]. The use of PEEP is beneficial to maintaining V/Q matching and improving oxygen supply, but it may also cause hemodynamic fluctuations. Michelet et al. [15] found that 5 cmH_2_O and 10 cmH_2_O levels of PEEP improved oxygenation, which was concordant with the findings of this study. This may be because of lung recruitment and no hemodynamic changes. However, some studies [27, 28] have shown that 4–5 cmH_2_O levels of PEEP during OLV can improve oxygenation, while increasing the PEEP level to 8–10 cmH_2_O did not improve oxygenation. This may be because the lungs were hyperinflated at this time and the high PEEP affected the hemodynamics. Hypoxemia defined by Schwarzkopf et al. [29] that a descent in arterial hemoglobin SaO_2_ to less than 90% did not occur in this study, which was probably related to the use of LPVS and high FiO_2_. Therefore, the application of PEEP with 5 cmH_2_O in thoracic surgery in elderly patients during OLV may be a wise choice, which avoids hyperinflation of the lung and has no significant hemodynamic fluctuations.

In this study, as shown in Figs. 3 and 4, the effect trend of PEEP on oxygenation and hemodynamic parameters can vary between patients and even in the opposite. This may be because PEEP affects many factors—it can affect cerebral blood flow perfusion by changing intrathoracic pressure and can also affect cardiac ejection and lung expansion and contraction—and ultimately presents a mixed effect. PEEP affects cardiac function mainly by altering vital capacity and intrathoracic pressure during ventilation. In our study, due to limitations in terms of the experimental conditions, we used the FloTrac/Vigileo apparatus to evaluate multiple hemodynamic parameters such as CI and SVV. There were no differences in CI between OLV and TLV, or among three PEEP levels during OLV in this study, which was in concordance with previous studies [25, 30]. The reason may be that cardiac output is affected by many factors, including preload, afterload, contractility, and ventricular compliance [31]. This study found that there was a significant difference in SVV between TLV and OLV because of the transpulmonary shunt. However, there was no difference in SVV among different levels of PEEP during OLV in this study, which is consistent with a previous study [32].

Now more studies have proposed the concept of optimal PEEP or individualized PEEP. Ferrando et al. [25] found that using individualized PEEP after an alveolar recruitment maneuver showed better oxygenation and lung mechanics improvements than a standardized 5 cmH_2_O of PEEP during OLV. A focus on individualized PEEP will be conducted in the next portion of the studies.

This study adopts a prospective randomized crossover-controlled method, a type of randomized controlled trial with a special self-controlled design. The main advantage is that the sample size can be significantly saved because subjects can receive multiple treatments; moreover, the method can also control the differences between individuals and the effect of time. For elderly patients receiving one-lung ventilation, we can choose the most favorable mode of ventilation to help them reduce complications. Moreover, for operations with a high incidence of complications such as lobectomy, anesthesiologists should play an important role in the perioperative period [33]. We need to make a careful preoperative assessment and coordinate with other relevant doctors to make a detailed case discussion. We should ensure patients’ safety and comfort during and after surgery and reduce the risk of complications.

One of the limitations of this study is that we were unable to perform Doppler ultrasonography of the brain. Measuring cerebral blood flow or cerebral blood flow velocity may further explain the decrease in rSO_2_ during OLV. The term " the elderly” generally refers to adults aged over 65 years. A study has revealed that people over 60 years of age show a decline in cerebral oxygenation and hemodynamics in the left prefrontal cortex [34], so we included patients aged 60 to 65 years. Another limitation is the application of 100% FiO_2_ perhaps may enhance the incidence of reabsorption atelectasis. We used 100% FiO_2_ in this study to avoid hypoxemia during OLV, considering the poor tolerance of hypoxemia in elderly individuals. The application of lower FiO_2_ may alter the differences in oxygenation observed among groups. At last, this study did not follow up on the patients for postoperative delirium, postoperative cognitive dysfunction, and other complications. We are actively following up on these aspects and hope that more studies can be performed to explain the clinical significance of low rSO_2_ on postoperative complications soon.

## Conclusions

In conclusion, the application of PEEP with 10 cmH_2_O makes rSO_2_ decrease compared with 0 cmH_2_O. Combining the results of this study, applying PEEP with 5 cmH_2_O during OLV in elderly patients can improve oxygenation and maintain high rSO_2_ levels, without significantly increasing peak airway pressure compared to not using PEEP.

### Electronic supplementary material

Below is the link to the electronic supplementary material.

Supplementary Material 1

## Data Availability

The datasets used and/or analyzed during the current study are available from the corresponding author upon reasonable request.

## Abbreviations

rSO_2_: Regional cerebral oxygen saturation
PEEP: Positive end-expiratory pressure
OLV: One-lung ventilation
PaO_2_: Arterial oxygen partial pressure
FiO_2_: Fraction of inspiration oxygen
Ppeak: Peak airway pressure
V/Q: Ventilation/Perfusion
PPCs: Postoperative pulmonary complications
LPVS: Lung protective ventilation strategy
ASA: American Society of Anesthesiologists
SpO_2_: Saturation of peripheral oxygen
BIS: Bispectral Index
VCV: Volume-controlled ventilation
VT: Tidal volume
PBW: Predicted Body Weight
RR: Respiratory rate
I: E: Inspiratory-to-expiratory
.P_ET_CO_2_: Pressure of end-tidal CO_2_
HR: Heart rate
MAP: Mean arterial pressure
CI: Cardiac index
SVV: Stroke volume variability
TLV: Two-lung ventilation
PaCO_2_: Arterial carbon dioxide partial pressure
ICP: Intracranial pressure
CVP: Central venous pressure
CPP: Cerebral perfusion pressure
CO: Cardiac output
CBF: Cerebral blood flow
SaO_2_: Arterial oxygen saturation
