# Supplementary material for: Effects of positive end-expiratory pressure on regional cerebral oxygen saturation in elderly patients undergoing thoracic surgery during one-lung ventilation: a randomized crossover-controlled trial
Source: BMC Pulm Med. 2024 Mar 6;24:120. doi: 10.1186/s12890-024-02931-z (PMC10919006; doi:10.1186/s12890-024-02931-z)
Supplement: Supplementary file 1 — Supplementary Material 1 [file 12890_2024_2931_MOESM1_ESM.docx]

Effects of Positive End-Expiratory Pressure on Regional Cerebral Oxygen Saturation in Elderly Patients Undergoing Thoracic Surgery during One-Lung Ventilation: A Randomized Crossover-Controlled Trial

*SUPPLEMENTARY DATA*

CATALOGUE

[Supplemental Methods 2](#_Toc156423853)

[S1.1 Vital Signs 2](#_Toc156423854)

[S1.2 Study Design 2](#_Toc156423855)

[S1.3 Data Collection 2](#_Toc156423856)

[S1.4 Primary and Secondary Outcomes 3](#_Toc156423857)

[S1.5 Computational Formulas 3](#_Toc156423858)

[eTables 4](#_Toc156423859)

[eTable 1. Validation of effects between groups of different orders. 4](#_Toc156423860)

[eTable 2. Characteristics of data which used repeated measures ANOVA. 8](#_Toc156423861)

[eTable 3. Pairwise Comparisons. 8](#_Toc156423862)

[eTable 4. Comparison of rSO_2_ lower than 65% in three groups of PEEP. 10](#_Toc156423863)

[eFigures 12](#_Toc156423864)

[eFigure 1. Protocol of study design. 12](#_Toc156423865)

[eFigure 2. Effects of PEEP on blood gas analysis, respiratory variables, and hemodynamics during thoracoscopic lobectomy. 12](#_Toc156423866)

Supplemental Methods

S1.1 Vital Signs

Maintain the patient's nasopharyngeal temperature at 36-37℃. During the operation, the peak airway pressure (Ppeak) should be lower than 30 cmH_2_O. If exceeded, the tidal volume should be gradually reduced by 1ml/kg each time until the Ppeak can be accepted. Keep intraoperative cardiac index (CI)≥2.0L/(min·m^2^), if it decreased, vasoactive drugs such as dopamine should be used as appropriate. Besides, keep stroke volume variability (SVV)≤13%; if it increases, appropriate fluids can be added. In the event of cerebral oxygen desaturation during surgery [regional cerebral oxygen saturation (rSO_2_) was reduced by 15% relative to baseline or rSO_2_ < 55%], the following measures were used: 1) pay attention to mechanical reasons caused by head position, electrode pad placement, or compression of blood vessels; 2) observe whether the mean arterial pressure (MAP) is severely reduced, and apply vasoactive drugs to adjust blood pressure; 3) if caused by hypocapnia, restore arterial carbon dioxide partial pressure (PaCO_2_) to normal; 4) if severe anemia was present, transfuse red blood cell based on blood gas results; 5) if the above interventions did not work, suppress brain metabolism (additional propofol or moderate cooling).

S1.2 Study Design

We used a prospective randomized crossover-controlled study method in this study. Every subject was applied positive end-expiratory pressure (PEEP) of three different levels successively: 0 cmH_2_O, 5 cmH_2_O, and 10 cmH_2_O. Patients were randomly assigned to one of six PEEP sequence combinations of 0, 5, and 10 cmH_2_O, and the specific sequence combinations were as follows: 1) 0 cmH_2_O, 5 cmH_2_O, 10 cmH_2_O; 2) 0 cmH_2_O, 10 cmH_2_O, 5 cmH_2_O; 3) 5 cmH_2_O, 0 cmH_2_O, 10 cmH_2_O; 4) 5 cmH_2_O, 10 cmH_2_O, 0 cmH_2_O; 5) 10 cmH_2_O, 0 cmH_2_O, 5 cmH_2_O; 6) 10 cmH_2_O, 5 cmH_2_O, 0 cmH_2_O.

S1.3 Data Collection

First, we collected data including the baseline rSO_2_ of two sides, heart rate (HR), blood pressure (BP), oxygen saturation (SpO_2_), peak airway pressure (Ppeak), the mean arterial pressure (MAP), cardiac index (CI), stroke volume variability (SVV) and arterial blood gas values 10 minutes after starting ventilation during TLV in a supine position. The arterial blood gas values covered arterial oxygen partial pressure (PaO_2_), and arterial carbon dioxide partial pressure (PaCO_2_). And calculated PaO_2_/Fraction of inspiration oxygen(FiO_2_) (P/F ratio) based on received PaO_2_ and pre-set FiO_2_. Then, measurements were performed three times respectively from 20 minutes after the first, second, and third PEEP application after One-lung ventilation (OLV).

The rSO_2_, arterial blood gases, ventilatory, and hemodynamics data were recorded at the following study points:

1. TLV: 10 min after placing the patient in the supine position and starting ventilation during Two-lung ventilation (TLV), finishing anesthetic induction.
2. OLV(0): 20 min after starting OLV ventilation with 0 cmH_2_O PEEP.
3. OLV(5): 20 min after starting OLV ventilation with 5 cmH_2_O PEEP.
4. OLV(10): 20 min after starting OLV ventilation with 10 cmH_2_O PEEP.

S1.4 Primary and Secondary Outcomes

The primary outcome was defined as the average of bilateral rSO_2_ among three different PEEPs at 20 minutes after adjusting every PEEP in OLV. And the secondary outcomes were as follows: 1) the average rSO_2_ between TLV and OLV; 2) hemodynamics parameters like HR, MAP, CI, and SVV between TLV and OLV, and among three PEEP in OLV ; 3) pulmonary oxygenation variables, such as PaO_2_, PaO_2_/FiO_2_, and Ppeak between TLV and OLV, and among three PEEP in OLV; 4) arterial blood gas values between TLV and OLV, and among three PEEP in OLV.

S1.5 Computational Formulas

Predicted body weight (PBW) was calculated using the Devine formula[1, 2], based on patient height and gender. For males = 50 kg +0.91×[height(cm)-152.4] kg , and for females = 45.5 kg +0.91×[height(cm)-152.4)] kg.

**References**

[1]. Pai, M.P. and F.P. Paloucek, The origin of the "ideal" body weight equations. Ann Pharmacother, 2000. 34(9): p. 1066-9.

[2]. Xu, Q., et al., Effects of dynamic individualized PEEP guided by driving pressure in laparoscopic surgery on postoperative atelectasis in elderly patients: a prospective randomized controlled trial. BMC Anesthesiol, 2022. 22(1): p. 72.

eTables

eTable 1. Validation of effects between groups of different orders.

| Groups | rSO_2_ | | | | PaO_2_ | | | |
| --- | --- | --- | --- | --- | --- | --- | --- | --- |
|  | Shapiro-Wilk test  *p* value | mean ± SD or median(IQR), % | Test of Homogeneity of Variances  *p* value | *P* value^a^ | Shapiro-Wilk test | mean ± SD or median(IQR), % | Test of Homogeneity of Variances  *p* value | *p* value^a^ |
|  |  |  |  |  | *p* value |  |  |  |
| 1 | 0.296 | 66.11±6.53 | NA | 0.077 | 0.108 | 210.78±108.30 | NA | 0.236 |
| 2 | 0.383 | 66.31±13.90 |  |  | 0.003 | 142.50(81.00,227.50) |  |  |
| 3 | 0.071 | 66.33±7.74 |  |  | 0.001 | 111.50(77.75,272.50) |  |  |
| 4 | 0.006 | 68.75(64.25,71.88) |  |  | 0.036 | 192.00(110.50,312.00) |  |  |
| 5 | 0.67 | 71.03±4.50 |  |  | 0.007 | 144.00(88.75,200.75) |  |  |
| 6 | 0.424 | 65.17±7.68 |  |  | 0.149 | 159.39±60.35 |  |  |
| Groups | PaCO_2_ | | | | Ppeak | | | |
|  | Shapiro-Wilk test  *p* value | mean ± SD,(mmHg) | Test of Homogeneity of Variances  *p* value | *p* value^b^ | Shapiro-Wilk test | mean ± SD or median(IQR),(cmH_2_O) | Test of Homogeneity of Variances  *p* value | *p* value^a^ |
|  |  |  |  |  | *p* value |  |  |  |
| 1 | 0.802 | 39.22±3.81 | 0.016 | 0.005 | 0.701 | 20.67±3.36 | NA | 0.605 |
| 2 | 0.713 | 38.39±2.50 |  |  | 0.864 | 21.39±3.07 |  |  |
| 3 | 0.992 | 39.11±2.91 |  |  | 0.306 | 22.22±2.56 |  |  |
| 4 | 0.099 | 38.33±3.20 |  |  | 0.007 | 21.00(19.75,25.00) |  |  |
| 5 | 0.373 | 42.78±5.29 |  |  | 0.081 | 21.61±2.66 |  |  |
| 6 | 0.851 | 41.89±3.83 |  |  | 0.285 | 20.94±2.44 |  |  |
| Groups | HR | | | | MAP | | | |
|  | Shapiro-Wilk test  *p* value | mean ± SD, (beats/min) | Test of Homogeneity of Variances  *p* value | *p* value^b^ | Shapiro-Wilk test | mean ± SD,(mmHg) | Test of Homogeneity of Variances  *p* value | *p* value^b^ |
|  |  |  |  |  | *p* value |  |  |  |
| 1 | 0.25 | 62.89±9.26 | 0.013 | 0.007 | 0.402 | 83.80±10.87 | 0.15 | 0.232 |
| 2 | 0.419 | 70.11±12.12 |  |  | 0.282 | 87.56±10.64 |  |  |
| 3 | 0.516 | 66.83±9.36 |  |  | 0.044 | 84.28±10.04 |  |  |
| 4 | 0.089 | 56.83±6.96 |  |  | 0.201 | 85.24±12.88 |  |  |
| 5 | 0.479 | 60.78±9.45 |  |  | 0.086 | 79.06±8.39 |  |  |
| 6 | 0.426 | 67.17±15.85 |  |  | 0.208 | 82.37±8.05 |  |  |
| Groups | CI | | | | SVV | | | |
|  | Shapiro-Wilk test  *p* value | mean ± SD or median(IQR),(L/min∙m^2^) | Test of Homogeneity of Variances  *p* value | *p* value^a^ | Shapiro-Wilk test  *p* value | mean ± SD or median(IQR),% | Test of Homogeneity of Variances  *p* value | *p* value^a^ |
| 1 | 0.765 | 2.29±0.49 | NA | 0.161 | 0.424 | 6.83±2.43 | NA | 0.068 |
| 2 | 0.237 | 2.04±0.24 |  |  | 0.347 | 7.28±2.65 |  |  |
| 3 | 0.361 | 2.06±0.40 |  |  | 0.793 | 9.44±2.87 |  |  |
| 4 | 0.12 | 2.01±0.30 |  |  | 0.061 | 7.94±2.10 |  |  |
| 5 | 0.095 | 2.37±0.57 |  |  | 0.005 | 6.00(5.00,9.50) |  |  |
| 6 | 0.003 | 2.20(1.98,2.48) |  |  | 0.001 | 6.00(5.00,9.25) |  |  |

^a^. Use the Kruskal Wallis test to analyze the data; ^b^. Use repeated measures ANOVA to analyze the data; NA, not applicable; SD, standard deviation; IQR, interquartile range; TLV, two-lung ventilation; OLV, one-lung ventilation; rSO_2_, regional cerebral oxygen saturation; PaO_2_, arterial oxygen partial pressure; PaCO_2_, arterial carbon dioxide partial pressure; Ppeak, peak airway pressure; HR, heart rate; MAP, mean arterial pressure; CI, cardiac index; SVV, stroke volume variability.

eTable 2. Characteristics of data which used repeated measures ANOVA.

| Variable | Shapiro-Wilk test | Test of Homogeneity of Variances | | Mauchly's Test of Sphericity | |
| --- | --- | --- | --- | --- | --- |
|  | p value | Levene Test ^a^ | p value | p value | Greenhouse-Geisser  **ω** |
| rSO_2_(0) | 0.109 | 0.049 | 0.952 | 0.034 | 0.828 |
| rSO_2_(5) | 0.377 |  |  |  |  |
| rSO_2_(10) | 0.249 |  |  |  |  |
| rSO_2_(TLV) | 0.766 | 0.556 ^b^ | 0.645 ^b^ | 0.003 ^b^ | 0.737 ^b^ |

TLV, two-lung ventilation; rSO_2_, regional cerebral oxygen saturation.

1. Based on the mean.
2. The results among TLV and three different PEEP levels in OLV.

eTable 3. Pairwise Comparisons.

| Pairwise Comparisons | | | | | | |
| --- | --- | --- | --- | --- | --- | --- |
| Variable | | Mean Difference | Std.Error | p value ^a^ | 95% Confidence Interval for Difference ^a^ | |
|  |  |  |  |  | Lower Bound | Upper Bound |
| rSO_2_(0)^b^ | rSO_2_(5) | 2.722 | 0.972 | 0.053 | -0.024 | 5.469 |
|  | rSO_2_(10) | 2.889* | 0.820 | 0.008 | 0.573 | 5.204 |
| rSO_2_(5)^b^ | rSO_2_(10) | 0.167 | 0.641 | >0.999 | -1.645 | 1.978 |
| rSO_2_(TLV)^b^ | rSO_2_(0) | 5.667* | 1.103 | <0.001 | 2.552 | 8.781 |
|  | rSO_2_(5) | 8.389* | 1.284 | <0.001 | 4.763 | 12.015 |
|  | rSO_2_(10) | 8.556* | 1.124 | <0.001 | 5.381 | 11.730 |
| PaO_2_(0) | PaO_2_(5) | -62.639 * | 22.018 | 0.005 | -106.170 | -19.108 |
|  | PaO_2_(10) | -73.389 * | 22.490 | 0.001 | -117.852 | -28.925 |
| PaO_2_(5) | PaO_2_(10) | -10.750 | 26.912 | 0.690 | -63.957 | 42.457 |
| PaO_2_(TLV) | PaO_2_(0) | 319.889 * | 17.473 | <0.001 | 285.344 | 354.434 |
|  | PaO_2_(5) | 257.250 * | 22.887 | <0.001 | 212.002 | 302.498 |
|  | PaO_2_(10) | 246.500 * | 23.341 | <0.001 | 200.354 | 292.646 |
| PaCO_2_(0)^b^ | PaCO_2_(5) | -0.583 | 1.961 | >0.999 | -5.913 | 4.746 |
|  | PaCO_2_(10) | -6.333* | 1.961 | 0.011 | -11.663 | -1.004 |
| PaCO_2_(5)^b^ | PaCO_2_(10) | -5.750* | 1.961 | 0.027 | -11.079 | -0.420 |
| PaCO_2_(TLV)^b^ | PaCO_2_(0) | 0.417 | 1.601 | >0.999 | -3.935 | 4.768 |
|  | PaCO_2_(5) | -0.167 | 1.601 | >0.999 | -4.518 | 4.185 |
|  | PaCO_2_(10) | -5.917* | 1.601 | 0.003 | -10.268 | -1.565 |
| Ppeak(0) | Ppeak(5) | -1.083 | 0.550 | 0.051 | -2.170 | 0.003 |
|  | Ppeak(10) | -4.222 * | 0.464 | <0.001 | -5.140 | -3.304 |
| Ppeak(5) | Ppeak(10) | -3.139 * | 0.491 | <0.001 | -4.110 | -2.167 |
| Ppeak(TLV) | Ppeak(0) | -3.417 * | 0.490 | <0.001 | -4.386 | -2.448 |
|  | Ppeak(5) | -4.500 * | 0.516 | <0.001 | -5.520 | -3.480 |
|  | Ppeak(10) | -7.639 * | 0.424 | <0.001 | -8.477 | -6.801 |
| HR(0)^b^ | HR(5) | 9.333 | 4.760 | 0.324 | -3.604 | 22.271 |
|  | HR(10) | 8.417 | 4.761 | 0.489 | -4.521 | 21.354 |
| HR(5)^b^ | HR(10) | -0.917 | 4.761 | >0.999 | -13.854 | 12.021 |
| HR(TLV)^b^ | HR(0) | -3.583 | 3.887 | >0.999 | -14.147 | 6.980 |
|  | HR(5) | 5.750 | 3.887 | 0.862 | -4.814 | 16.314 |
|  | HR(10) | 4.833 | 3.887 | >0.999 | -5.730 | 15.397 |
| MAP(0) | MAP(5) | -3.019 | 2.524 | 0.234 | -8.009 | 1.972 |
|  | MAP(10) | -2.519 | 2.430 | 0.302 | -7.323 | 2.286 |
| MAP(5) | MAP(10) | 0.500 | 2.384 | 0.834 | -4.124 | 5.214 |
| MAP(TLV) | MAP(0) | 3.891 | 2.560 | 0.122 | -1.079 | 9.042 |
|  | MAP(5) | 0.963 | 2.516 | 0.702 | -4.011 | 5.937 |
|  | MAP(10) | 1.463 | 2.422 | 0.547 | -3.325 | 6.251 |
| CI(0) | CI(5) | 0.003 | 0.105 | 0.979 | -0.205 | 0.210 |
|  | CI(10) | 0.044 | 0.106 | 0.675 | -0.165 | 0.254 |
| CI(5) | CI(10) | 0.042 | 0.104 | 0.689 | -0.164 | 0.247 |
| CI(TLV) | CI(0) | 0.039 | 0.106 | 0.715 | -0.249 | 0.171 |
|  | CI(5) | -0.036 | 0.105 | 0.731 | -0.243 | 0.171 |
|  | CI(10) | 0.006 | 0.105 | 0.985 | -0.203 | 0.214 |
| SVV(0) | SVV(5) | 0.722 | 0.817 | 0.378 | -0.892 | 2.337 |
|  | SVV(10) | 0.778 | 0.706 | 0.273 | -0.619 | 2.174 |
| SVV(5) | SVV(10) | 0.056 | 0.825 | 0.946 | -1.576 | 1.687 |
| SVV(TLV) | SVV(0) | 1.306 * | 0.631 | 0.040 | 0.057 | 2.554 |
|  | SVV(5) | 2.028 * | 0.762 | 0.009 | 0.522 | 3.534 |
|  | SVV(10) | 2.083 * | 0.642 | 0.001 | 0.814 | 3.353 |

TLV, two-lung ventilation; OLV, one-lung ventilation; rSO_2_, regional cerebral oxygen saturation; PaO_2_, arterial oxygen partial pressure; PaCO_2_, arterial carbon dioxide partial pressure; Ppeak, peak airway pressure; HR, heart rate; MAP, mean arterial pressure; CI, cardiac index; SVV, stroke volume variability.

^*^ The mean difference is significant at the 0.05 level.

1. Adjustment for multiple comparisons.
2. Use Bonferroni analysis for pairwise comparisons. Others use the generalized linear mixed model.

eTable 4. Comparison of rSO_2_ lower than 65% in three groups of PEEP.

| Variable |  | PEEP |  | Statistic | | *P* | |
| --- | --- | --- | --- | --- | --- | --- | --- |
|  | OLV(0) | OLV(5) | OLV(10) | |  |  |  |
| rSO_2_, n (%) |  |  |  |  | |  |  |
| ＜65 % | 11 (30.56) | 17 (47.22) | 14 (38.89) | χ²=2.104 | | 0.349 |  |
| ≥65 % | 25 (69.44) | 19 (52.78) | 22 (61.11) |  |  |  |  |

rSO_2_, regional cerebral oxygen saturation; OLV, one-lung ventilation; OLV(0), OLV ventilation with 0 cmH_2_O PEEP; OLV(5), OLV ventilation with 5 cmH_2_O PEEP; OLV(10), OLV ventilation with 10 cmH_2_O PEEP.

eFigures

eFigure 1. Protocol of study design.


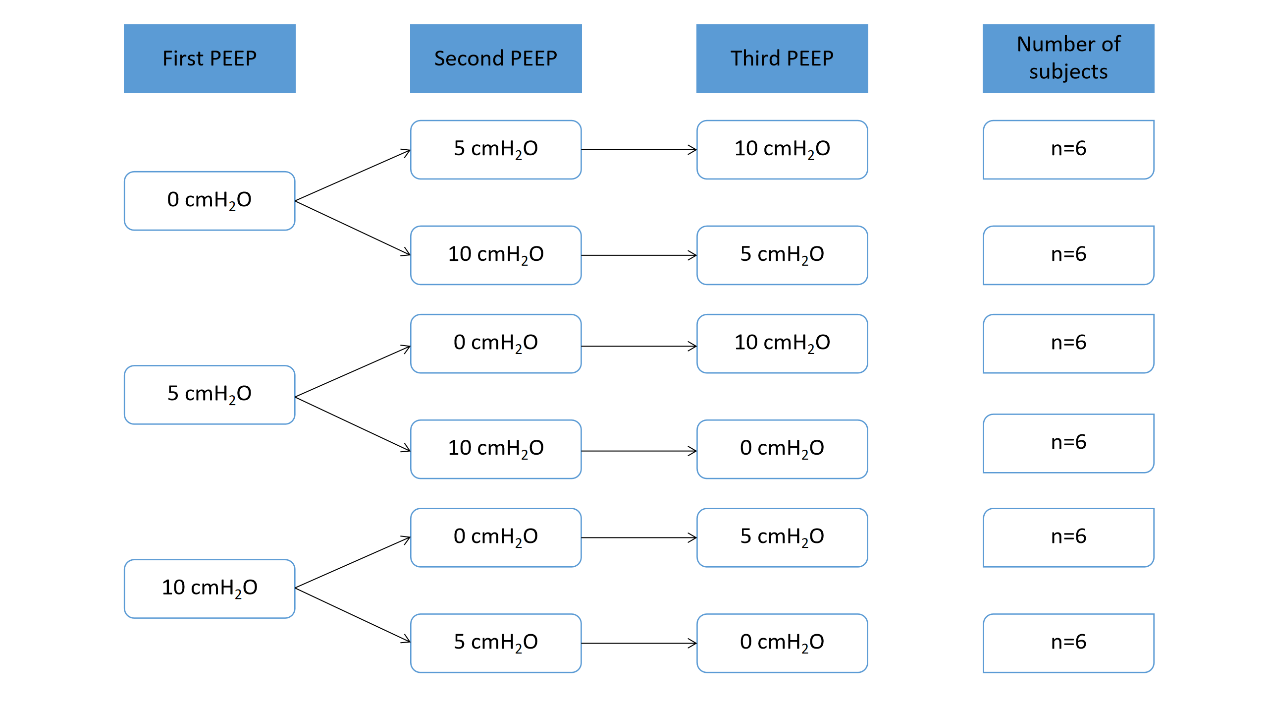


eFigure 2. Effects of PEEP on blood gas analysis, respiratory variables, and hemodynamics during thoracoscopic lobectomy.

(A.) PaCO_2_ at TLV was statistically lower than OLV(10), *p*=0.003. * OLV(0) vs OLV(10): *p*=0.011; ** OLV(5) vs OLV(10): *p*=0.027.

(B.) There were statistical differences among TLV and three PEEP levels in Ppeak. * OLV(0) vs OLV(10): *p*＜0.001 ** OLV(5) vs OLV(10): *p*＜0.001.

(C.)-(E.) There were no statistically significant differences between TLV and OLV, or among different PEEP groups of OLV.

(F.) There were statistical differences among TLV and three PEEP levels of OLV in SVV. No significant differences among the three PEEPs in OLV.

TLV, two-lung ventilation; OLV, one-lung ventilation; OLV(0), OLV ventilation with 0 cmH_2_O PEEP; OLV(5), OLV ventilation with 5 cmH_2_O PEEP; OLV(10), OLV ventilation with 10 cmH_2_O PEEP; PaCO_2_, arterial carbon dioxide partial pressure; Ppeak, peak airway pressure; HR, heart rate; MAP, mean arterial pressure; CI, cardiac index; SVV, stroke volume variability.

**
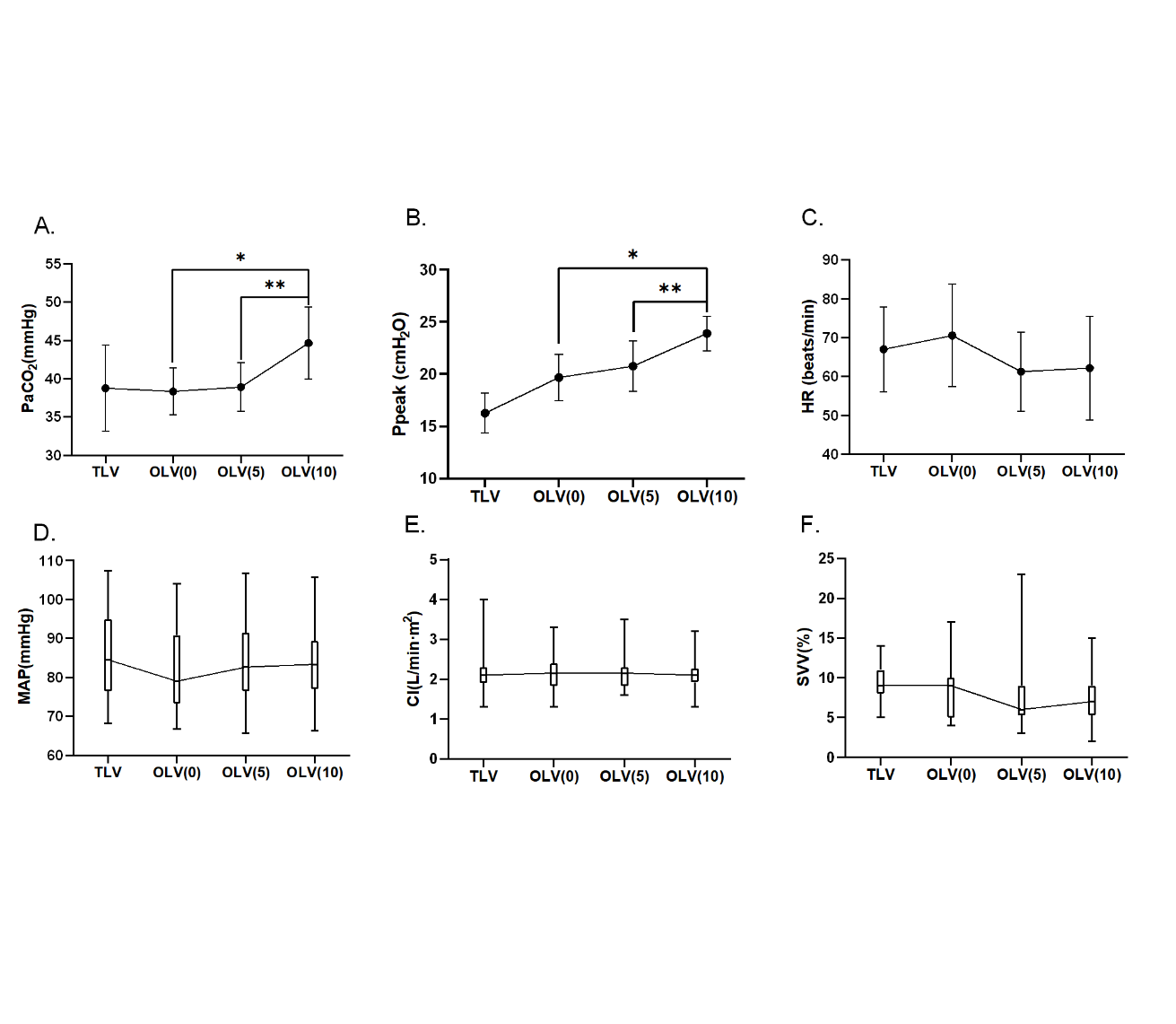
**
